# Nanoparticles as Efflux Pump and Biofilm Inhibitor to Rejuvenate Bactericidal Effect of Conventional Antibiotics

**DOI:** 10.1186/s11671-017-2222-6

**Published:** 2017-07-13

**Authors:** Divya Gupta, Ajeet Singh, Asad U. Khan

**Affiliations:** 1 Medical Microbiology and Molecular Biology Lab., Interdisciplinary Biotechnology Unit, Aligarh Muslim University, Aligarh, 202002 India; 2 Department of Biotechnology, Mangalayatan University, Aligarh, 202145 India; 3 Department of Biotechnology, G. B. Pant Engineering College, Pauri, 246194 India

**Keywords:** Efflux pumps, Metallic nanoparticles, Quorum-sensing biomolecules, Anti-biofilm, Conventional antibiotics, Synergy

## Abstract

The universal problem of bacterial resistance to antibiotic reflects a serious threat for physicians to control infections. Evolution in bacteria results in the development of various complex resistance mechanisms to neutralize the bactericidal effect of antibiotics, like drug amelioration, target modification, membrane permeability reduction, and drug extrusion through efflux pumps. Efflux pumps acquire a wide range of substrate specificity and also the tremendous efficacy for drug molecule extrusion outside bacterial cells. Hindrance in the functioning of efflux pumps may rejuvenate the bactericidal effect of conventional antibiotics. Efflux pumps also play an important role in the exclusion or inclusion of quorum-sensing biomolecules responsible for biofilm formation in bacterial cells. This transit movement of quorum-sensing biomolecules inside or outside the bacterial cells may get interrupted by impeding the functioning of efflux pumps. Metallic nanoparticles represent a potential candidate to block efflux pumps of bacterial cells. The application of nanoparticles as efflux pump inhibitors will not only help to revive the bactericidal effect of conventional antibiotics but will also assist to reduce biofilm-forming capacity of microbes. This review focuses on a novel and fascinating application of metallic nanoparticles in synergy with conventional antibiotics for efflux pump inhibition.

## Review

The chronic infections identified with biofilms are difficult to eradicate as they are able to resist both antibiotics as well as the host immune system [1]. Biofilm barrier is one of the main reasons for conversion of acute to chronic infections [2]. As indicated by the report of the national institute of health and center of disease control, approximately 65–80% diseases are caused through biofilm-inducing bacteria, predominantly through Gram-negative bacteria *Pseudomonas aeruginosa* and *Escherichia coli* and Gram-positive bacterium *Staphylococcus aureus* [3]. Antibiotics have been appearing to be ineffective in treating infections having the biofilm, on account of their limited capacity to cross the biofilm rampart and to extirpate the targeted bacterial cells [4]. Additionally, bacteria have evolved a unique efflux system to drain out toxic substances and waste products outside the bacterial cell [5]. Efflux pumps are membrane-bound transporter proteins having a wide spectrum of substrate specificity and immense drug exclusion capacity [6].

All these diseases that associated concerns pertinent to biofilm and efflux pumps lead to the emergence of multidrug-resistant (MDR) bacteria or extensive drug-resistant (EDR) bacteria; owing to this, nanoparticles in conjunction with conventional antibiotics have been proposed as an alternative approach to eradicate or damage biofilm as well as to treat MDR or EDR infections.

These new antimicrobials, metallic nanoparticles, not only enhance the antimicrobial activity of existing antibiotics but also revive their bactericidal activity. The synergistic application of antibiotics and metallic nanoparticles exhibited more of their potential antimicrobial effect rather than their individual application [7, 8]. The utilization of nanoparticles with antibiotics as anti-biofilm or efflux pump inhibitor has been well examined and explored [1, 9–11]. Metallic nanoparticles have been extensively used to treat infections in human cell lines due to their low cytotoxicity (concentration dependent), high surface area, and broad-spectrum antibacterial activity [12–14]. Moreover, the combined application of metallic nanoparticles with an antibiotic reduces their concentration as drug dosage and hence diminishes the toxicity of both agents to human cell lines [15]. This review emphasizes the synergistic application of nanoparticles with antibiotics as anti-biofilm and efflux pump inhibitor on which extensive research has been carried out to combat infections caused by MDR or EDR pathogens.

## Nanoparticles as Efflux Pump Inhibitors

Various studies have been carried out to determine the mode of action of nanoparticles as a bactericidal agent. However, the several points on the mechanism of inhibitory action of nanoparticles on microorganisms still remain to be resolved. One of the possible mechanisms for bactericidal activity of nanoparticles is attributed to the inhibition of efflux pumps. Banoee et al. in 2010 adduce a novel efflux pump inhibitory role of zinc oxide nanoparticles on NorA efflux pumps of *S. aureus*. They have discovered 27 and 22% increase in the zone of inhibition for ciprofloxacin in the presence of zinc oxide nanoparticles in *S. aureus* and *E. coli*, respectively [16]. Afterward, Padwal et al. in 2014 propound the concept of synergistic use of polyacrylic acid-coated iron oxide (magnetite) nanoparticles (PAA-MNP) with rifampicin against *Mycobacterium smegmatis* with emphasis on the efflux inhibitory role of PAA-MNP. They have used a fusion of PAA-MNP and rifampicin in *M. smegmatis* which resulted in fourfold higher growth inhibition in contrast with rifampicin alone. It may be explained through threefold increased accumulation of antibiotic inside bacterial cells as proven with real-time transport studies on a common efflux pump substrate, ethidium bromide [17].

There are two possible mechanisms available through which metal nanoparticles can impede the working of efflux pumps. One possible mechanism is the direct binding of metal nanoparticles to the active site of efflux pumps, blocking the extrusion of antibiotics outside the cells. Metal nanoparticles may here act as a competitive inhibitor of antibiotic for the binding site of efflux pumps [18]. Another possible mechanism is through the disruption of efflux kinetics. The effect of silver nanoparticles for disruption of the efflux kinetics of MDR efflux pump, MexAM-OPrM, has already been examined in *P. aeruginosa* [19]. It may be suggested that metal nanoparticles may lead to termination of proton gradient followed by disruption of membrane potential or loss of proton motive force (PMF), resulting in deterioration of driving force essential for efflux pump activity [18, 20, 21]. However, the major constraint in the direct binding of nanoparticles with efflux pumps is their small size and reactivity. Additionally, nanoparticles may also bind with other membrane proteins rather than interacting simply with efflux pumps, and because of that, the chance of nanoparticles associating particularly with an efflux transporter each time amid the exposure is restricted.

Christena et al. has shown earlier in their studies regarding efflux inhibitory role of copper nanoparticles on the NorA efflux pump, partly due to the generation of Cu(II) ions from copper nanoparticles. This partial effect directly from copper nanoparticles may imply the direct interaction of nanoparticles with efflux pumps, supporting the first hypothesis, while partial effect due to release of Cu(II) ions might indicate the disruption of membrane potential and perturbing working of efflux pumps, supporting the second hypothesis [9]. Chatterjee et al. have also revealed the loss of membrane potential of *E. coli* cells from −185 to −105 and −75 mV after growing bacterial cells in the presence of 3.0 and 7.5 μg/ml concentration of copper nanoparticles, respectively, for 1 h [22]. The explicit mechanism for the efflux inhibitory role of nanoparticles still remains puzzling and requires further research.

## Nanoparticles as Anti-Biofilm Agent

Biofilm provides resistance to bacteria, but this defiance gets intensified if biofilm is produced by drug-resistant bacteria [23]. Numerous studies have shown tremendous capabilities of metal nanoparticles to disintegrate thick biofilm barrier through the various modes of actions [24–27]. The penetrating power of metallic nanoparticles always remains a utile feature to employ them against biofilm infections [28–30]. This unique amalgamation of two diverse modalities, nanoparticles and antibiotic, paved a new way to combat against biofilm producing MDR or EDR bacteria.

One of the eloquent studies was conducted by Gurunathan et al. to elucidate the augmented bactericidal and anti-biofilm effect of different antibiotics with silver nanoparticles. Symbiotic use of ampicillin and silver nanoparticles greatly enhanced the biofilm inhibition in Gram-negative and Gram-positive bacteria by 70 and 55%, respectively, in contrast with approximately 20% biofilm inhibition after treated with silver nanoparticles alone. Similarly, the combined application of silver nanoparticles and vancomycin results in 55 and 75% biofilm inhibition in Gram-negative and Gram-positive bacteria, respectively [10]. These results suggest the alternative use of nanoparticle with antibiotics to induce biofilm inhibition opening clinical possibilities of novel therapy.

A similar effect was also observed for copper nanoparticles and zinc oxide nanoparticles using synergistically with antibiotics. According to this study, unification of copper nanoparticles and antibiotics showed more effective anti-biofilm activity in contrast with zinc oxide nanoparticle and antibiotic combination in both Gram-positive as well as Gram-negative bacteria. This increased inhibition with copper nanoparticles may be because of extrusion of Cu(II) ions generated from nanoparticles. Copper nanoparticles and zinc oxide nanoparticles coupled with specific antibiotic have exhibited enhanced anti-biofilm effect in the presence of 2% glucose, revealing increased bonding interactions between metallic nanoparticles and antibiotic in the presence of glucose [9]. Coating of metal nanoparticles with carbohydrates may transmogrify nanoparticle-cell interaction, cellular uptake, and cytotoxicity [31].

## The Alliance Between Efflux Systems and Quorum Sensing

**Fig. 1:**
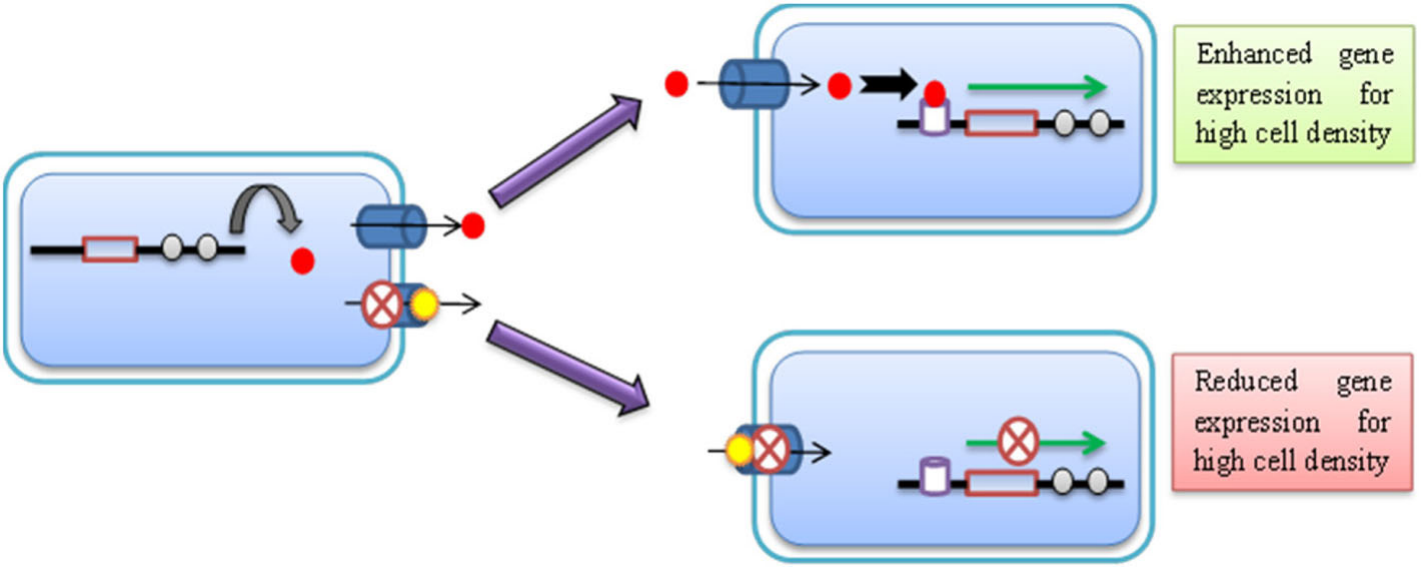
Use of metal nanoparticles as an efflux pump inhibitor to impede extrusion of quorum-sensing signaling molecules (*red filled circle*) outside bacterial cells with the help of metal nanoparticles (*yellow filled circle*) to block efflux pump (*filled cylinder*) resulting in reduced binding of signaling molecule to its receptor (*empty cylinder*) and hindrance in biofilm formation

Efflux pumps play an important role in cell to cell signaling of biomolecules to assist biofilm formation. One probable mechanism to combat drug resistance is to employ the efflux pump inhibitors to block the quorum-sensing mechanism, ultimately hindering biofilm formation. Several studies have already been conducted to show the role of efflux pumps in quorum sensing [23, 32–39]. The explicit mechanistic relationship between efflux pumps and biofilm formation is still not fully understood. One viable justification could be the role of efflux pumps to extrude critical components required in quorum sensing. This functional aspect has already been proposed in earlier studies [40–46]. Impairment in the extrusion of signaling molecule through the application of efflux pump inhibitors may affect the process of quorum sensing or cell to cell signaling [47, 48] (Fig. 1).

**Fig. 2:**
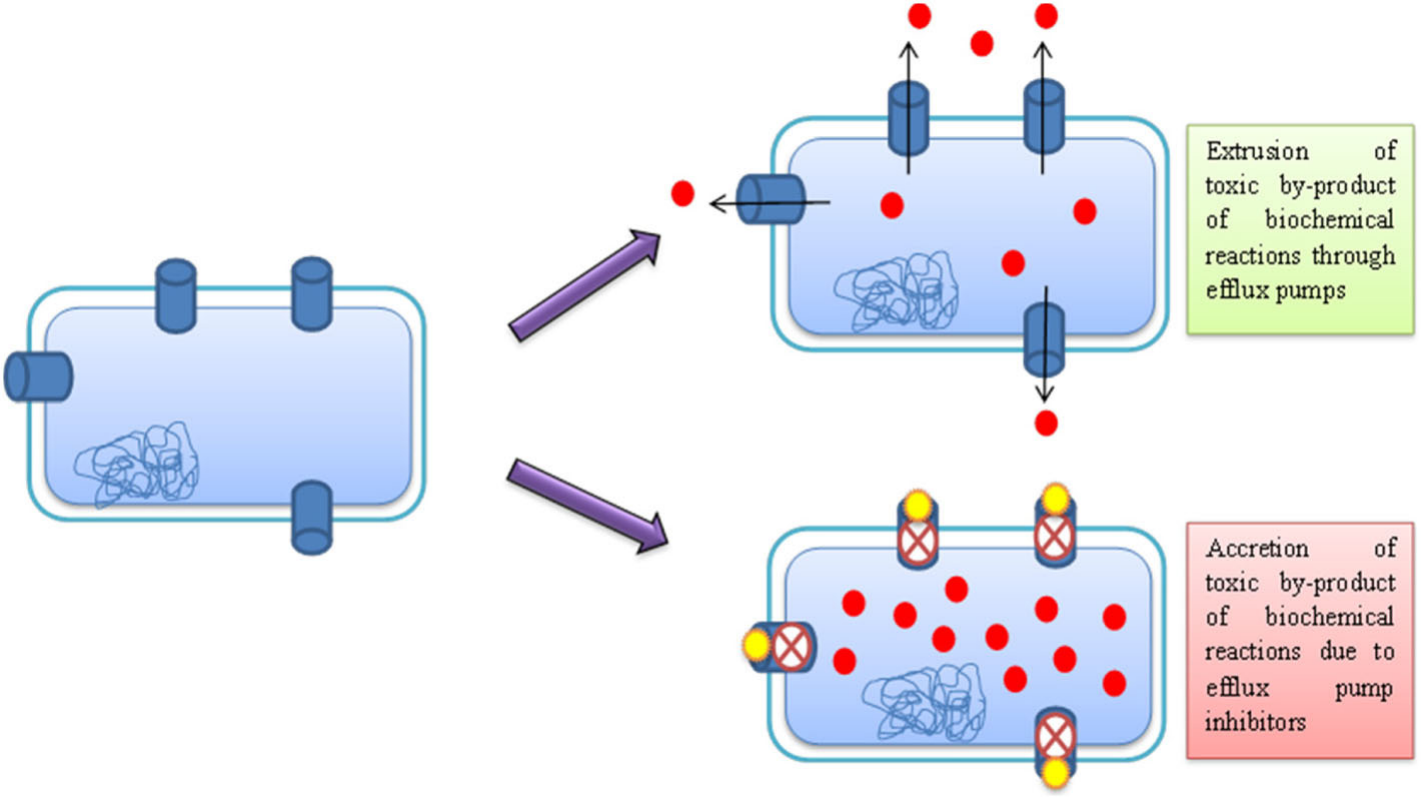
Use of metal nanoparticles as an efflux pump inhibitor to impede extrusion of toxic by-product of biochemical reactions (*red filled circle*) outside bacterial cells with the help of metal nanoparticles (*yellow filled circle*) to block efflux pump (*filled cylinder*) resulting in hindrance in biofilm formation

Another viable justification could be the role of efflux pumps to export toxic and waste by-product outside the cells. Rapidly metabolizing cells in the biofilm may rely upon the conductive system to extrude out the noxious and waste by-product resulting from various biochemical activities taking place inside bacterial cells [49]. This functional aspect has also been proposed in the study conducted by Kvist et al. in 2008 [50]. Employment of efflux pump inhibitors to block this conductive system may result in higher accretion of toxic by-product inside the bacterial cells, ultimately reducing biofilm formation (Fig. 2).

Some studies have also suggested the detriment of efflux pumps to affect cellular aggregation by transmuting cell membrane properties and ultimately affecting biofilm formation [47]. Numerous studies have already been conducted to deduce the effect of nanoparticles in combination with antibiotics as improved antibacterial and anti-biofilm agent. Table 1 contains a summary of these studies.

**Table 1:** Summary of the studies deducing synergy of nanoparticles and antibiotic as antimicrobial and anti-biofilm agent

Reference	Type of nanoparticle	Antibiotic	Species		Report year
			Gram-positive	Gram-negative	
52	Ag nanoparticle	Ampicillin, chloramphenicol, kanamycin	*Enterococcus faecium*, *Staphylococcus aureus*	*Pseudomonas aeruginosa*, *Escherichia coli*	2012
24	Ag nanoparticle	Gentamicin, ampicillin, ofloxacin, vancomycin	–	*Escherichia coli*	2013
10	Ag nanoparticle	Ampicillin, vancomycin	*Staphylococcus aureus*, *Streptococcus pneumoniae*	*Pseudomonas aeruginosa*, *Shigella flexneri*	2014
11	Cu nanoparticle	Ciprofloxacin	*Staphylococcus aureus*	*Pseudomonas aeruginosa*	2015
51	Ag nanoparticle	Amikacin, kanamycin, oxytetracycline, streptomycin	*Staphylococcus aureus*	*Pseudomonas aeruginosa*, *Escherichia coli*	2016
9	Cu nanoparticle, ZnO nanoparticle	Ceftriaxone, ceftazidime, gentamicin	*Enterococcus faecalis*, *Staphylococcus aureus*	*Pseudomonas aeruginosa*, *Escherichia coli*, *Shigella flexneri*, *Klebsiella pneumoniae*	2016

One of the recent studies has been conducted by Barapatre et al., deducing enhanced synergistic antibacterial and anti-biofilm activity of silver nanoparticles in combination with amikacin, kanamycin, oxytetracycline, and streptomycin antibiotic against Gram-positive and Gram-negative bacteria. Centralizing over green chemistry, silver nanoparticles were synthesized through the enzymatic reduction of silver nitrate by engaging two lignin-degrading fungus, viz., *Aspergillus flavus* and *Emericella nidulans*. It was suggested to use nanoparticles as a probe with conventional antibiotics to augment antibacterial and anti-biofilm activity against pathogenic microbes [51]. Disruption of ATP-dependent function like efflux pump inhibition has been reported as one of the potential mechanisms of synergistic effect of antibiotics and metallic nanoparticles [52].

A number of reports have successfully demonstrated appliance of nanoparticles against two diverse mechanisms of bacterial resistance, viz., MDR efflux pumps and biofilm formation, through which bacteria evade the action of conventional antibiotics. This review represents a new and promising approach to employ metallic nanoparticles in synergy with antibiotics as efflux pump inhibitor and anti-biofilm agent both to combat antibiotic resistance.

## Conclusions

In the current scenario, there is an urge for an innovative approach for controlling MDR infections. Efflux pumps play dual roles, one is to extrude out antibiotics and the other is to assist in biofilm formation through expelling biomolecules important for quorum sensing, ultimately contributing to the virulence of bacterial pathogens. The blockage of MDR efflux pumps through nanoparticles will be helpful in both directions; it blocks the efflux of antibiotic outside the bacterial cells and hence increasing the effect of conventional antibiotics, and also, it blocks the efflux of quorum-sensing biomolecules and hence decreasing biofilm-forming capacity of bacterial cells. This approach reduces the need to conduct new research to investigate novel efflux pump inhibitor or new antibiotic but encourages the use of metal nanoparticles (employing as an efflux pump inhibitor) in synergy with conventional antibiotics. It will also assist in reducing the cost, the time, and the cytotoxicity problem of nanoparticles in the human cell lines which can endure a lower concentration of metallic nanoparticles. It would be a novel approach to target efflux pumps, reducing quorum-sensing signals in order to suppress biofilm formation.

## Future Prospects

Bacterial evolution has resulted in the adoption of various mechanisms to revert the bactericidal effect of antibiotic and the host immune system. It leads to the generation of multidrug-resistant infections reflecting an urgent need to discover new approaches to fight against MDR or XDR infections. With the emergence of antibiotic resistance, symbiotic use of metallic nanoparticle with conventional antibiotics offers a better alternative for wrangling against antibiotic resistance. The application of nanoparticles as efflux pump inhibitors can be of great importance in two diversified directions but ending to one single outcome, i.e., to fight against bacterial infections. The exact mechanism of action of nanoparticles to block efflux pumps still needs to be investigated. It is likely that disruption of PMF could be a probable indirect mechanism by which nanoparticles might inhibit efflux. One of the major challenges with this approach is allied to the reactivity of nanoparticles that may render them to associate with other membrane proteins rather than efflux transporter proteins. This can be overcome by preparing targeted nanoparticles by linking them with anti-efflux monoclonal antibodies or lectins. This proper inhibition will calibrate them for focusing on a particular locale. Another considerable challenge may be the toxicity problem, using this approach owing to the large surface to volume ratio of small-sized nanoparticles which should be optimized before final validation.
